# Mitophagy and Noncoding RNA Regulation in Type 2 Diabetes Mellitus: Molecular Mechanisms, Tissue-Specific Evidence and Translational Perspective

**DOI:** 10.3390/biomedicines14091886

**Published:** 2026-08-24

**Authors:** Ashish Kothari, Mundakkassery Pullurmanna Narayanan, Shashi Ranjan Mani Yadav, Reena Kumari, Harsh Kumar, Radhika Kherdekar, Shivmurat Yadav, Pallab Shaw, Baskar Chakrapani, Prawej Ansari, Ankur Kumar, Shrinkhal, Dinesh K. Patel, Veronique Seidel, Atul Pandey, Anoop Misra, Sandeep Kumar

**Affiliations:** 1 Department of Ophthalmology and Visual Science, University of Michigan, MI 48105, USA; ashishkothari1212@gmail.com; 2 Department of Microbiology, All India Institute of Medical Sciences, Rishikesh 249203, India; ankurk591@gmail.com; 3 Department of Biochemistry, All India Institute of Medical Sciences, Rishikesh 249203, India; omnarayananmp@gmail.com (M.P.N.); ranjanshashi2011@gmail.com (S.R.M.Y.); drbaskar.bio@gmail.com (B.C.); 4 Department of Biochemistry, NAMO Medical Education and Research Institute, Silvassa 396230, India; 5 Department of Biochemistry, Swaminarayan Institute of Medical Sciences & Research, Gandhinagar 382725, India; 6 Department of Genetics, University of Alabama at Birmingham, Birmingham, AL 35294, USA; reena.xlnet@gmail.com (R.K.); chemist89ansari@gmail.com (P.A.); 7 Department of Cell and Molecular Biology, St. Jude Children’s Research Hospital, Memphis, TN 38105, USA; visitharsh@gmail.com; 8 Department of Dentistry, Periodontics, All India Institute of Medical Sciences, Rishikesh 249203, India; radhika96k@gmail.com; 9 Department of Medicine, University of Oklahoma Health Campus, Oklahoma City, OK 73104, USA; yshivmurat@gmail.com; 10 Centre for Diabetes Research, School of Biomedical Sciences, Ulster University, Coleraine BT52 1SA, UK; 11 Department of Ophthalmology, All India Institute of Medical Sciences, Raebareli 229405, India; shrinkhalbhu@gmail.com; 12 Department of Mechanical Engineering, Carnegie Mellon University, Pittsburgh, PA 15213, USA; dineshpa@andrew.cmu.edu; 13 Natural Products Research Laboratory, Strathclyde Institute of Pharmacy and Biomedical Sciences, University of Strathclyde, Glasgow G4 0RE, UK; veronique.seidel@strath.ac.uk; 14 Department of Ecology and Evolutionary Biology, Princeton University, Princeton, NJ 08536, USA; 15 Department of Endocrinology, Fortis Hospital, New Delhi 110048, India; anoopmisra@gmail.com; 16 Department of Microbiology/Immunology, Tulane University, New Orleans, LA 70112, USA

**Keywords:** mitochondrial quality control, PINK1/Parkin pathway, oxidative stress, insulin resistance, diabetic complications, mitochondrial dynamics, translational biomarkers

## Abstract

Despite significant therapeutic advances, T2DM remains a global public health challenge that leads to multiple complications, including cardiovascular, renal, hepatic, and neurodegenerative disorders. Mitochondrial dysfunction and impaired mitophagy remain fundamental, yet incompletely understood, mechanisms driving pancreatic β-cell failure, chronic inflammation, insulin resistance and diabetic complications. Emerging evidence indicates that noncoding RNAs (including microRNAs, long noncoding RNAs, and circular RNAs) are critical regulators of mitophagy and mitochondrial quality control mechanisms across metabolically active tissues. This review comprehensively examines the interplay between mitochondrial dysfunction, mitophagy impairment, and T2DM pathophysiology. It provides an overview of recent mechanistic insights into mitophagy–noncoding RNA interactions in T2DM, emphasizing tissue-specific effects, and highlights the translational potential of mitophagy-associated proteins and regulatory ncRNAs as diagnostic biomarkers and therapeutic targets. By bridging fundamental molecular biology with translational and clinical perspectives, further it provides a comprehensive framework to guide future research, accelerate biomarker discovery, and support the development of personalized interventions aimed at reducing the growing worldwide burden of T2DM and its complications.

## 1. Introduction

In 2024, it was estimated that approximately 589 million adults aged 20–79 years had diabetes. This was equivalent to about one in nine adults worldwide. This number is projected to increase to around 853 million by 2050 [1]. Diabetes mellitus (DM) is a metabolic disorder characterized by hyperglycemia and disruptions in carbohydrate, protein, and fat metabolism. There are three main types of DM: type 1 diabetes, type 2 diabetes, and gestational diabetes mellitus (GDM). In type 1 diabetes, the autoimmune destruction of pancreatic β-cells results in absolute insulin deficiency. On the other hand, type 2 diabetes mellitus (T2DM), which accounts for approximately 90% of all cases, involves impaired insulin function leading to insulin resistance (IR), often accompanied by varying degrees of insulin secretion deficiency. T2DM is common among middle-aged and older adults, although obesity, physical inactivity, and other metabolic risk factors contribute to its increasing incidence in younger populations. GDM occurs when there is glucose intolerance during pregnancy and usually resolves after delivery. More than 80% of adults with diabetes live in low- and middle-income countries, highlighting the need for affordable diagnostic and therapeutic approaches [2,3,4].

Insulin regulates glucose homeostasis by facilitating glucose uptake in peripheral tissues and reducing gluconeogenesis. In DM, impaired insulin secretion or action leads to inefficient glucose metabolism, promoting gluconeogenesis, glycogenolysis, and lipolysis while suppressing glycolysis and lipogenesis [5]. The resulting chronic hyperglycemia, dyslipidemia, elevated circulating fatty acids, and low-grade inflammation together sustain metabolic stress that drives disease progression [6]. If left untreated, DM leads to severe complications, such as neuropathy, cardiovascular disease, nephropathy, and retinopathy [7].

At the cellular level, Type 1 and Type 2 DM are associated with alterations in mitochondrial function, although the underlying mechanisms and affected tissues differ between the two types [8]. Mitochondria play an important role in cellular processes, such as metabolism, redox balance, apoptosis, calcium homeostasis, endoplasmic reticulum stress (ERS) response, inflammasome activation, and autophagy [9,10,11]. Mitophagy is a selective form of autophagy through which damaged or dysfunctional mitochondria are recognized, sequestered, and degraded. In cultured cells and animal models, defective mitophagy has been associated with hyperglycemia, IR, and β-cell stress [6]. Dysfunctional mitochondria and disruptions in mitophagy have also been implicated in the pathophysiology of aging [10], cardiovascular disease [11], cancer [12], obesity and metabolic syndrome [13] as well as neurodegenerative disorders [11,14,15,16]. In this review, the aforementioned conditions were only considered if they provided insight into shared mitochondrial mechanisms, metabolic risk factors, or complications directly relevant to T2DM.

Noncoding RNAs (ncRNAs) are regulatory transcripts that influence gene expression at the transcriptional, post-transcriptional, and epigenetic levels and that participate in processes relevant to insulin production, secretion, sensitivity, and cellular stress responses. They have been reported to regulate pancreatic β-cell differentiation and survival, insulin-signaling cascades in peripheral tissues, glucose and lipid metabolism, inflammation, oxidative stress, ERS response, and vascular, renal, and neural function [17,18,19]. In addition to these metabolic functions, selected microRNAs (miRNAs), long noncoding RNAs (lncRNAs), and circular RNAs (circRNAs) have been shown to influence mitochondrial biogenesis, mitochondrial fission and fusion, oxidative stress, and mitophagy. These effects involve regulation of the PINK1/Parkin, BNIP3/NIX, FUNDC1, AMPK-mTOR-ULK1, and sirtuin-related pathways [20,21]. Mitophagy and ncRNAs should therefore be considered interconnected rather than separate processes: dysregulated ncRNA expression can alter mitochondrial quality-control pathways, while mitochondrial and metabolic stress can, in turn, alter ncRNA expression, creating tissue- and context-dependent regulatory loops. This review further explores the role of mitophagy and ncRNAs in human health, with a special focus on T2DM.

Previous reviews have frequently examined mitochondrial dysfunction and mitophagy in T2DM separately from the broader roles of ncRNAs or have focused on individual tissues and diabetic complications. The present narrative review brings these areas together within a tissue-specific framework encompassing pancreatic β-cells, liver, skeletal muscle, adipose tissue, kidney, heart, and vascular tissue. It also distinguishes findings obtained from cell-based experiments, animal models, human tissues, and circulating samples. Special emphasis is given to the direct regulation of mitophagy-associated pathways by ncRNAs, and to the current limitations of these molecules as biomarkers and therapeutic targets in T2DM [22].

## 2. Literature Search Strategy

This narrative review critically analyzes the literature on mitochondrial dysfunction, mitophagy, and noncoding RNA regulation in T2DM and its complications. Relevant publications were retrieved by searching PubMed and Google Scholar. The search terms included those related to T2DM, mitochondrial quality control, and noncoding RNAs: “type 2 diabetes mellitus,” “insulin resistance,” “pancreatic beta cell” or “pancreatic β-cell,” “mitochondrial dysfunction,” “mitochondrial quality control,” “mitophagy,” “mitophagic flux,” “ PTEN-induced kinase 1 (PINK1),” “Parkin” or “PRKN,” “BCL2 interacting protein 3 (BNIP3),” “NIX” or “BCL2 interacting protein 3 like (BNIP3L),” “FUN14 domain-containing protein 1 (FUNDC1),” “AMP-activated protein kinase, AMPK,” “mechanistic target of rapamycin, mTOR,” “Unc-51-like autophagy-activating kinase 1, ULK1,” “mitochondrial fission,” “mitochondrial fusion,” “DRP1,” “noncoding RNA,” “microRNA” or “miRNA,” “long noncoding RNA” or “lncRNA,” and “circular RNA” or “circRNA.” These terms were used singly and in combination with tissue- and complication-specific terms such as “liver,” “skeletal muscle,” “adipose tissue,” “kidney,” “heart,” “vascular endothelium,” “retina,” “neuropathy,” “diabetic nephropathy,” “diabetic cardiomyopathy,” and “diabetic retinopathy.”

No strict lower date limit was applied to publications, because seminal studies were required to describe the discovery and molecular basis of mitochondrial quality-control pathways. Priority was given to recent publications related to T2DM, mitophagy, ncRNA regulation, and diabetic complications. Only relevant peer-reviewed publications in English scoping the scientific literature from 1988 to 2026 were retrieved. The most recent literature search was performed on 17 August 2026. Reference lists of relevant reviews and primary research articles were also examined to identify additional studies not captured in the initial search.

Studies were included if they focused on mitophagy, mitochondrial dynamics, mitochondrial quality control, or ncRNA regulation in T2DM, IR, obesity-induced metabolic dysfunction, or diabetic complications; (ii) investigated defined molecular pathways or targets, including PINK1/Parkin, BNIP3/NIX, FUNDC1, AMPK-mTOR-ULK1, sirtuin-related pathways, or mitochondrial fission–fusion proteins; (iii) provided evidence from human subjects, human tissues, animal models, or experimentally relevant cell systems; (iv) evaluated the therapeutic or biomarker relevance of mitophagy-associated proteins or ncRNAs. Studies on aging, cardiovascular disease, cancer, and other non-diabetic conditions were only included if they described a mitochondrial mechanism, metabolic risk factor, or tissue response of direct relevance to T2DM.

Publications were excluded if they (i) were unrelated to mitochondrial quality control or ncRNA regulation; (ii) lacked a clear link to T2DM or its complications; (iii) were duplicate evidence already presented in more informative studies; (iv) made mechanistic claims without sufficient experimental support. For this purpose, a “more informative” study was defined as one providing substantially overlapping evidence but with a more complete methodological description, a larger or better-characterized study population, additional experimental validation, or more direct mechanistic evidence relevant to the specific topic being discussed. This criterion was applied only when the evidence was substantially overlapping; independent studies providing distinct mechanistic, tissue-specific, clinical, or translational information were retained. Similarity in study conclusions alone was not considered sufficient grounds for exclusion.

Conference abstracts, unpublished data, and non-peer-reviewed preprints were excluded. Non-peer-reviewed preprints were excluded irrespective of whether peer-reviewed evidence addressing the same topic was available. When a preprint identified during the literature search had subsequently been published as a peer-reviewed article, only the final peer-reviewed version was considered.

All retrieved articles were qualitatively analyzed and categorized by their molecular mechanism, tissue type, study model, relevance to β-cell dysfunction, IR, and diabetic complications. Because this work was conducted as a narrative rather than a systematic review, database retrieval and screening counts were not prospectively recorded during the initial literature search. To improve transparency, a retrospective verification of the literature included in the review was performed on 17 August 2026. The 243 records represented in the manuscript reference list were re-screened against the stated eligibility criteria. One duplicate publication was identified, resulting in 242 relevant publications that were assessed for eligibility and retained in the final narrative synthesis. During this verification, the reference list was also checked for consistency with the stated exclusion of non-peer-reviewed preprints.

## 3. Mitochondrial Dysfunction in Diabetes

Mitochondria are double-membrane organelles and the primary producers of ATP (the main source of energy for cells) through oxidative phosphorylation. They are also involved in other biological processes, including apoptosis, aging, autophagy, calcium homeostasis, amino acid and lipid metabolism, signaling, and reactive oxygen species (ROS) production [23]. The process of mitochondrial quality control ensures that mitochondrial functions are efficiently regulated. This involves the exchange, segregation and removal of damaged mitochondrial components [24,25,26,27]. Increased mitochondrial ROS production can disrupt mitochondrial function [26]. Other sources of intracellular ROS production include xanthine oxidase, lipoxygenases, cyclooxygenases, nicotinamide adenine dinucleotide phosphate oxidases, nitric oxide synthases, and peroxidases [28]. Within mitochondria, complexes I and III of the electron transport chain are major sites of superoxide generation, although the relative contribution of mitochondria to total cellular ROS varies according to cell type, metabolic state, and experimental conditions [29,30]. Damaged mitochondria are particularly susceptible to oxidative stress and produce more ROS and less ATP, thereby contributing to cellular dysfunction [27,31].

The chronic metabolic stress observed in diabetes (hyperglycemia, lipotoxicity, and excessive ROS production) impairs mitochondrial membrane potential and ATP production, reduces insulin secretion, and ultimately promotes apoptosis. Studies of pancreatic islets obtained from patients with T2DM have demonstrated abnormal mitochondrial morphology, altered membrane potential, reduced ATP production, and impaired glucose-stimulated insulin secretion. Most of the molecular mechanisms linking glucotoxicity, lipotoxicity, and mitochondrial injury to β-cell failure have been defined primarily in cultured cells and animal models [32]. In insulin-responsive tissues such as skeletal muscle, liver, and adipose tissue, nutrient excess and chronic hyperglycemia compromise mitochondrial function and increase ROS, which interferes with insulin-signaling pathways and contributes to IR, a central feature of T2DM [33,34]. Together with chronic hyperglycemia, dyslipidemia, inflammation, endothelial dysfunction, and vascular injury, IR contributes to the development of nephropathy, neuropathy, retinopathy, hypertension, and cardiovascular disease [35,36,37]. As the nature and extent of mitochondrial dysfunction differ among pancreatic β-cells, skeletal muscle, liver, adipose tissue, kidney, heart, and vascular tissues, this emphasizes the need for tissue-specific interpretation of the available evidence. Among the mitochondrial quality-control mechanisms affected by these metabolic stresses, mitophagy is particularly important for the selective removal of dysfunctional mitochondria.

## 4. The Role of Mitophagy in Diabetes

The protective role of autophagy in maintaining cellular homeostasis has been well-documented [22,38]. For instance, in response to ERS, autophagy degrades aggregated, unfolded/misfolded proteins and damaged endoplasmic reticulum (ER) components, thus restoring cellular homeostasis and limiting excessive ROS generation. Autophagy is not centrally regulated by mitochondrial ROS; instead, it is controlled by interconnected nutrient- and energy-sensing pathways, particularly AMPK and mTOR, and is further influenced by insulin signaling, ERS, hypoxia, and oxidative stress [39,40]. Beyond restoring ER and mitochondrial function, autophagy helps eliminate irreversibly damaged macromolecules and activates the transcription factor nuclear factor erythroid 2-related factor 2 (Nrf2), thereby promoting detoxification through antioxidant gene expression [41].

Although autophagy is a well-studied mechanism with known benefits for cells, its exact impact on T2DM remains unclear and appears to depend on specific tissue types [42]. Clinical studies do not support a uniform reduction in autophagy across all insulin-responsive tissues [43]. It has been observed that in skeletal muscle biopsies from patients with T2DM, the abundance of autophagy- and mitophagy-related genes and proteins was comparable to that in lean and obese individuals without diabetes [44]. In contrast, increased autophagy-related activity has been reported in adipose tissue from obese individuals with T2DM [45,46]. Since insulin suppresses autophagy, the hyperinsulinemia present in diabetic patients who have not yet developed IR may explain the downregulation of autophagic proteins observed in skeletal muscle, while in adipose tissue, the increase in autophagy varies depending on the type of tissue, the presence of obesity, and IR [47,48]. In the liver, autophagy appears to be reduced in the presence of IR and hyperinsulinemia [49]. Overall, the precise role of autophagy/mitophagy in the primary insulin-target tissues (muscle, adipose tissue, liver) in the context of diabetes and IR remains unclear.

Mitophagy, a specialized autophagic process that selectively removes damaged or dysfunctional mitochondria, is crucial for maintaining mitochondrial integrity [26,50]. Mitophagy also helps cells adapt from normoxic to hypoxic conditions by reducing mitochondrial numbers. These mechanisms are important for several cellular processes, including erythrocyte maturation and elimination of paternal mitochondria during mammalian fertilization [51,52,53,54]. In energy-demanding tissues like the brain, skeletal muscles, heart, liver, and kidney, mitochondria are highly specialized to balance energy supply and demand, a process tightly regulated by mitophagy [27].

In the context of T2DM, studies have revealed that peripheral blood mononuclear cells from diabetic patients had a significantly reduced expression of mitophagy markers compared with those from prediabetic individuals [55,56]. Current evidence suggests that chronic hyperglycemia and the ensuing IR characteristic of T2DM suppress autophagy. This inhibition is likely to stem from the oxidative stress and mitochondrial dysfunction associated with diabetes [55,57,58]. It has been reported that in metabolic conditions like diabetes, the dysregulation of canonical PINK1/Parkin-mediated mitophagy (alongside receptors such as BNIP3/NIX) increases mitochondrial dysfunction and ROS production [58,59]. In the liver, skeletal muscle, and adipose tissue, defective mitophagy contributes to mitochondrial fragmentation, impaired fatty-acid oxidation, elevated ROS, and disrupted organ-specific metabolism [60,61]. In adipocytes, excess mitochondrial clearance triggers inflammatory lipolysis and the release of free fatty acids, which in turn fuel hepatic steatosis and IR [62,63,64]. In liver cells, mitochondrial dysfunction disrupts gluconeogenesis and promotes ectopic lipid accumulation [65]. In skeletal muscle, dysfunctional mitochondria reduce glucose uptake and oxidation, fostering IR and hyperglycemia [66]. Collectively, these organ-specific defects in mitophagy promote systemic hyperglycemia, IR, and β-cell stress, driving the progression of T2DM.

**Figure 1:**
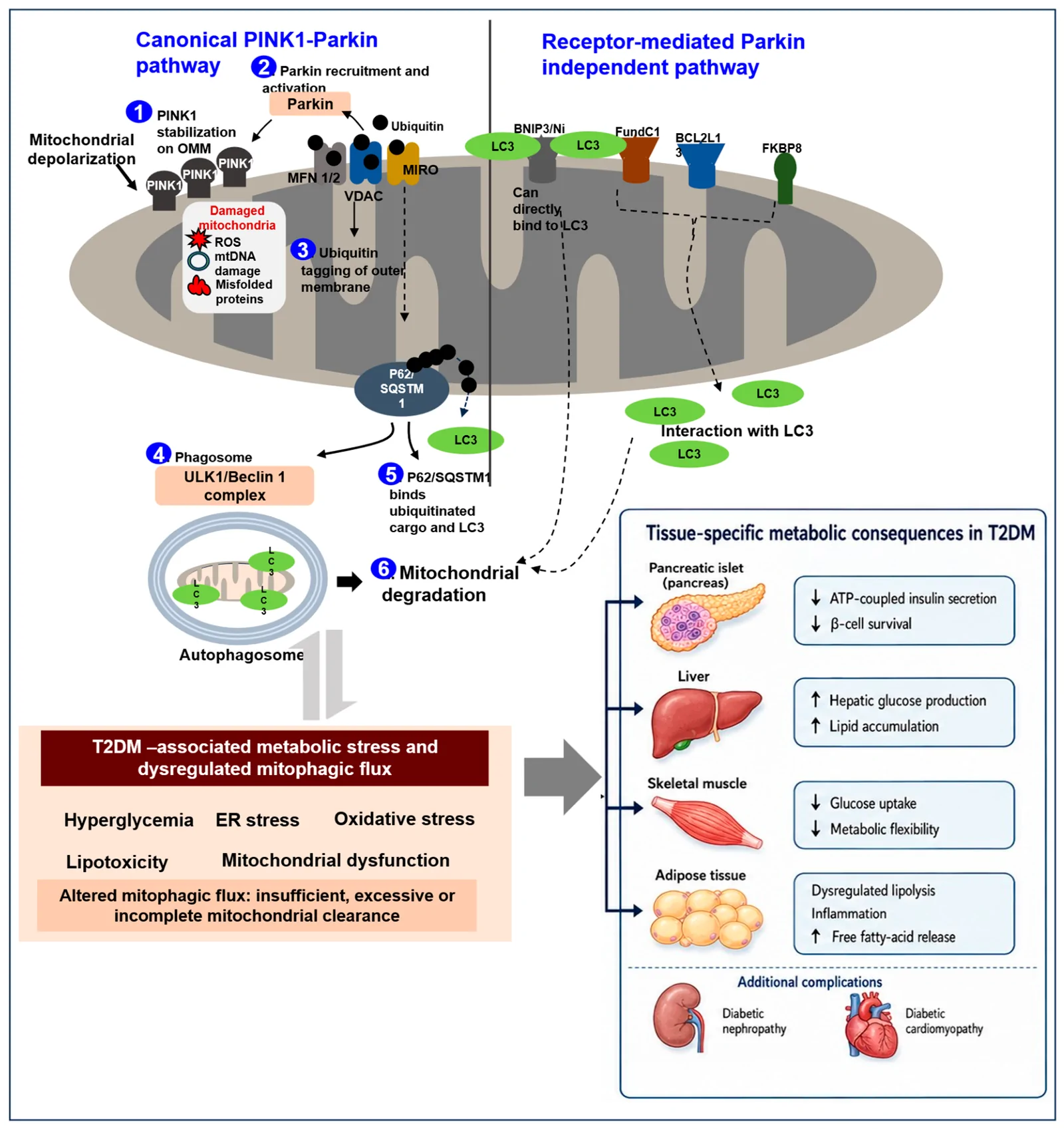
Tissue-dependent metabolic effects of canonical and receptor-mediated mitophagy pathways in type 2 diabetes mellitus. Mitochondrial depolarization results in stabilization of PINK1 on the outer mitochondrial membrane (OMM), promoting recruitment and activation of Parkin. Parkin ubiquitinates outer-membrane proteins such as mitofusin 1/2 (MFN1/2), voltage-dependent anion channel (VDAC), and mitochondrial Rho GTPase 1 (MIRO1). The adaptor protein p62/sequestosome 1 (p62/SQSTM1) recognizes ubiquitinated mitochondrial cargo, interacts with microtubule-associated protein 1 light chain 3 (LC3), and promotes the sequestration of damaged mitochondria within autophagosomes initiated by the ULK1-Beclin 1 complex. Moreover, mitochondria can be targeted via Parkin-independent receptor-mediated pathways such as BNIP3/NIX, FUNDC1, BCL2L13, and FKBP8, which directly or indirectly interact with LC3 to promote mitochondrial clearance. Hyperglycemia, lipotoxicity, ERS, oxidative stress, and mitochondrial dysfunction may impair mitophagic flux in T2DM, resulting in insufficient, excessive, or incomplete clearance of damaged mitochondria. These alterations have tissue-specific effects, including reduced ATP-coupled insulin secretion and impaired β-cell survival in pancreatic islets, increased hepatic glucose production and lipid accumulation in the liver, reduced glucose uptake and metabolic flexibility in skeletal muscle, and dysregulated lipolysis, inflammation, and free-fatty-acid release in adipose tissue. Impaired mitochondrial quality control mechanisms also play a role in diabetic nephropathy and diabetic cardiomyopathy. The figure highlights that mitophagy in T2DM is not uniformly impaired, but rather dysregulated in a tissue- and context-dependent manner.

The impact of dysregulated mitochondrial turnover is not uniform across tissues; rather follows organ-specific patterns. Within pancreatic β-cells, the disruption of mitochondrial quality control mechanisms undermines ATP-dependent insulin release and jeopardizes cell viability. In liver cells, impaired autophagy/mitophagy triggers ER stress and lipid buildup. Within skeletal muscles, the compromised mitochondrial turnover is associated with diminished metabolic flexibility and impaired substrate oxidation, though data from clinical studies are conflicting. In adipose tissue, increased autophagic activity along with inflammation and adipocyte dysfunction can occur. Preclinical evidence has implicated defective mitophagy in the pathogenesis of diabetic nephropathy and diabetic cardiomyopathy, although confirmation in human studies is still limited. Taken together, these tissue-specific mitophagic alterations may collectively contribute to systemic hyperglycemia, IR, and β-cell dysfunction. However, current evidence points toward a model of dysregulated mitophagy, rather than a globally suppressed process, as the underlying feature of T2DM (Figure 1). Consequently, differentiating among adaptive, insufficient, and excessive mitophagic responses is essential when evaluating mitophagy-associated proteins as potential biomarkers or therapeutic targets. The major tissue-specific defects in mitophagy and mitochondrial quality control, together with their mitochondrial and metabolic consequences, are summarized in Table 1 and illustrated in Figure 1.

## 5. Signaling Pathways of Mitophagy in Diabetes

**Figure 2:**
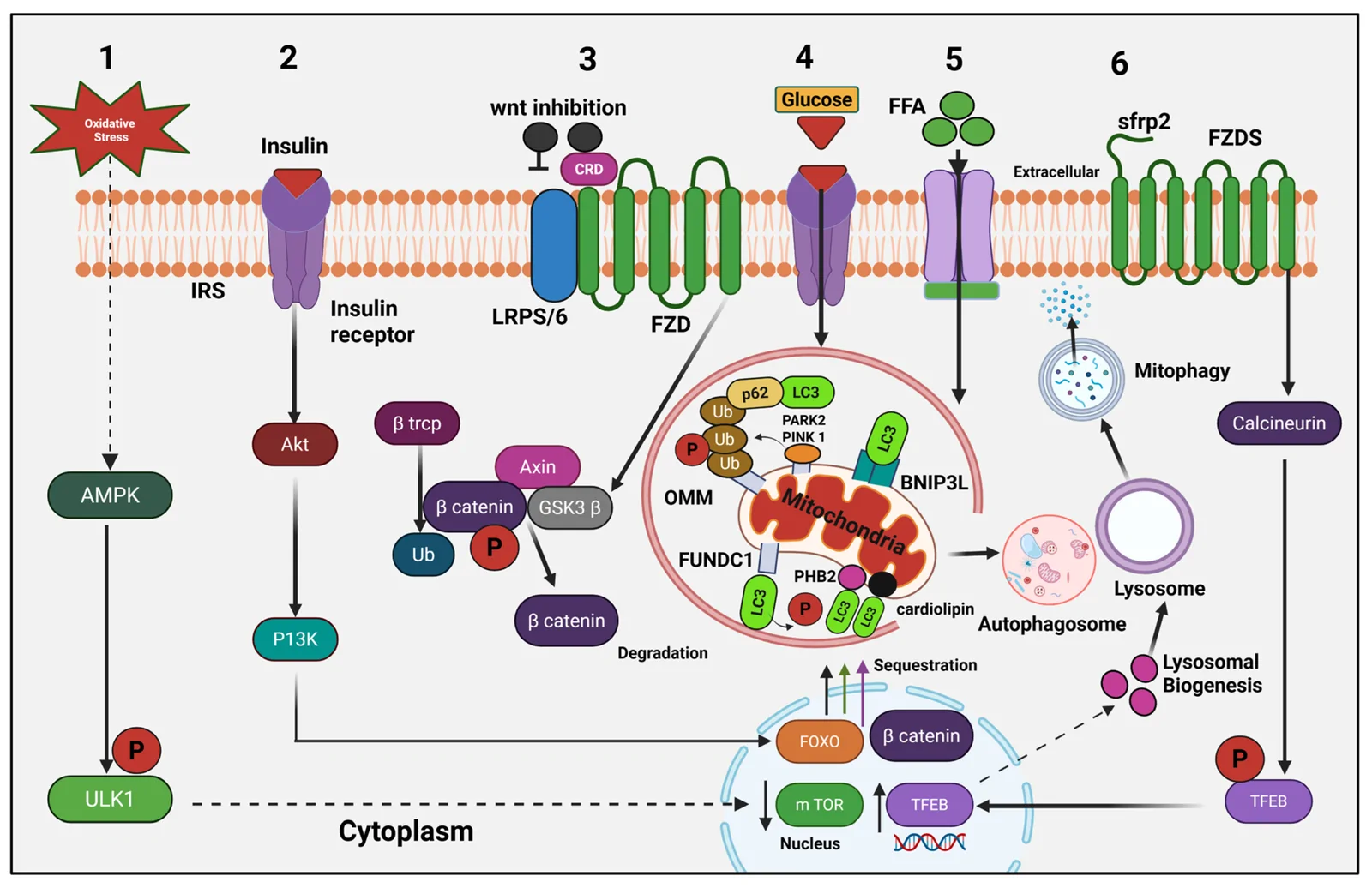
Key signaling pathways involved in regulation of mitophagy and lysosomal function in type 2 diabetes mellitus and diabetic cardiomyopathy. The schematic summarizes interconnected nutrient-, insulin-, Wnt-, glucose-, and stress-responsive pathways that regulate mitochondrial quality control. (1) Oxidative and energetic stress activate AMPK, which phosphorylates and activates ULK1 to initiate autophagy and mitophagy. (2) Insulin receptor signaling through PI3K and AKT influences ULK1, mTOR, and FOXO-dependent transcription; disruption of this pathway in IR may indirectly impair autophagy and mitophagy. (3) Canonical Wnt signaling via Frizzled/LRP receptors inhibits the Axin GSK3β/β-TrCP destruction complex, leading to stabilization and subsequent nuclear translocation of β-catenin. Under oxidative stress, β-catenin can also bind FOXO family transcription factors to regulate metabolic and stress-response programs. (4) Glucose- and nutrient-sensing signals converge on mitochondria and the autophagic machinery, where ubiquitinated mitochondrial proteins are recognized by p62/SQSTM1 and LC3, promoting sequestration of damaged mitochondria into autophagosomes. (5) Secreted frizzled-related protein 2 (SFRP2) signaling via Frizzled-5 can trigger a calcineurin–TFEB axis that promotes TFEB nuclear translocation, lysosomal biogenesis, autophagosome–lysosome fusion, and mitophagic clearance. (6) Calcineurin dephosphorylates TFEB to further stimulate its nuclear localization and the transcription of lysosomal and autophagy-related genes. Together, these pathways regulate the balance between mitochondrial damage, autophagosome formation, lysosomal degradation, and mitochondrial turnover. Dysregulation of these processes under hyperglycemic, lipotoxic, and oxidative-stress conditions may underlie impaired mitophagy, mitochondrial dysfunction, inflammation, and cardiac injury in T2DM. The SFRP2–FZD5-calcineurin–TFEB axis is based largely on preclinical evidence and requires confirmation in patients with diabetic cardiomyopathy.

The various signaling pathways driving mitophagy at the molecular level, including potential targets for mitophagy control, are illustrated in Figure 2. The latter takes diabetic cardiomyopathy as an example.

### 5.1. AMPK-mTOR Pathway

The AMPK is a conserved energy sensor that regulates glucose and lipid metabolism to maintain cellular energy homeostasis [78]. In metabolically active tissues, AMPK is activated when cellular ATP levels fall or ADP levels rise. AMPK dysfunction has been implicated in T2DM and associated metabolic syndromes, characterized by reduced glucose uptake and mitochondrial biogenesis, which contribute to IR [79]. In T2DM, sustained nutrient overload and dysregulated cellular energy homeostasis impair the normal crosstalk between AMPK and mechanistic target of rapamycin complex 1 (mTORC1). ULK1, the mammalian ortholog of yeast Atg1, serves as a key initiating kinase of autophagy and contributes to the regulation of mitophagy. When nutrients are abundant, mTORC1 associates with the ULK1 complex and phosphorylates both ULK1 and Atg13, thereby suppressing autophagic activity. In particular, phosphorylation of ULK1 at Ser757 by mTORC1 weakens the ULK1–AMPK interaction. Conversely, during nutrient scarcity or energy stress, mTORC1 dissociates from the ULK1 complex, resulting in enhanced ULK1 activity. Activated ULK1 subsequently phosphorylates Atg13 and FIP200 to trigger autophagy initiation. In parallel, AMPK can directly phosphorylate ULK1, providing an additional route by which autophagy is promoted [46]. ULK1 also links general autophagy initiation to selective mitochondrial clearance. During hypoxia or mitochondrial depolarization, ULK1 can translocate to damaged mitochondria and phosphorylate FUNDC1, thereby increasing its interaction with LC3 and promoting mitophagy [80]. Thus, the AMPK-mTORC1-ULK1 pathway allows mitochondrial turnover to respond to changes in cellular energy and nutrient availability. However, this mechanism has been defined mainly in experimental systems, and its tissue-specific regulation in patients with T2DM remains poorly understood.

### 5.2. Insulin Signaling Pathway

Studies have established a strong connection between autophagy, mitophagy, and IR [81]. In experimental models, the accumulation of damaged mitochondria increases mitochondrial ROS and releases mitochondrial danger signals that promote activation of the NLRP3-ASC-caspase-1 inflammasome. NLRP3 activation contributes to the production of inflammatory cytokines and has been linked to obesity-associated inflammation and IR [82]. However, this evidence does not establish impaired mitophagy as the sole cause of inflammasome activation in patients with T2DM. Conversely, the removal of damaged mitochondria reduces oxidative and inflammatory stress in experimental models, but there is currently insufficient clinical evidence to conclude that increasing mitophagy alone can alleviate diabetes. Insulin signaling regulates metabolism and autophagy through the IRS-PI3K-AKT-FOXO pathway [83,84]. Under nutrient-replete conditions, insulin-mediated activation of PI3K-AKT stimulates mTORC1 and inhibits FOXO transcription factors, thereby reducing autophagy initiation and the transcription of several autophagy-related genes. FOXO3 regulates genes such as LC3 and BNIP3, providing a possible link between insulin signaling, autophagy, and mitochondrial turnover [85]. ULK1, a key regulator of autophagy, is inhibited by AKT and mTORC1 but activated by AMPK [46]. Additionally, insulin inhibits autophagy during early life [42]. Several mitophagy-associated proteins are involved in the insulin signaling pathway. For instance, decreased Miro1 levels in T2DM reduce mitophagy and impair β-cell function by blocking the IRS-AKT-FOXO1 pathway, leading to decreased glucose tolerance [86].

### 5.3. Wnt-Related Signaling and the SFRP2-FZD5-TFEB

In early human development, the canonical Wnt/β-catenin pathway regulates pluripotency and cell fate in stem cells [87]. Wnt ligands, secreted glycoproteins, bind to Frizzled receptors and LRP5/6, forming a complex that inhibits GSK3-mediated phosphorylation. This inhibition reduces the degradation of ubiquitinated β-catenin, leading to its accumulation in the cytoplasm and subsequent translocation to the nucleus, where it interacts with LEF/TCF to regulate the expression of target genes such as c-Myc and cyclin D1 [88,89]. Another protein in this pathway, GSK3, is involved in glucose metabolism and is often dysregulated in metabolic disorders [90,91]. In diabetes, increased ROS redirect the limited β-catenin pool from TCF/LEF-mediated transcription toward FOXO-mediated transcription, contributing to decreased insulin sensitivity [92,93]. A more direct Wnt-related mechanism has recently been described in experimental diabetic cardiomyopathy: in glucolipotoxic H9c2 cells and diabetic rats, secreted frizzled-related protein 2 (SFRP2) interacted with Frizzled-5 (FZD5) and activated the calcineurin–transcription factor EB (TFEB) pathway, thereby increasing mitophagy and lysosomal activity and reducing cardiac injury. Notably, SFRP2 did not alter β-catenin levels in this study, suggesting that its effect was independent of canonical Wnt/β-catenin signaling [94]. These findings are relevant to Figure 2 but remain preclinical and have not yet been confirmed in patients with diabetic cardiomyopathy. The principal mitophagy pathways and upstream metabolic signaling networks, including their key regulators, tissue-specific evidence, and functional consequences in diabetes, are summarized in Table 2.

## 6. Aging as a Modifier of Mitophagy and T2DM Risk

The role of mitophagy in aging and age-related diseases remains incompletely understood, despite autophagy being recognized as a key quality-control mechanism linked to aging. Available experimental evidence suggests that mitophagy must be coordinated with mitochondrial biogenesis to preserve a functional mitochondrial population during aging [112]. In the Caenorhabditis elegans model, age-related disruption of this coordination resulted in the accumulation of damaged mitochondria, reduced stress resistance, and impaired cellular function [113]. However, direct evidence demonstrating an equivalent mechanism in aging human tissues remains limited. Aging is accompanied by the accumulation of somatic mitochondrial DNA (mtDNA) mutations, deletions, and heteroblastic variants. As mtDNA alterations accumulate with age, mitochondrial respiration and function decline, particularly once a functionally harmful variant expands beyond a tissue-specific threshold [114,115]. Age-related reductions in mitochondrial oxidative capacity and mitochondrial quality control may decrease metabolic flexibility and increase susceptibility to IR and pancreatic β-cell dysfunction. In older adults, reduced skeletal-muscle mitochondrial oxidative activity has been associated with lipid accumulation and IR, although this does not establish defective mitophagy as the primary cause [116]. Therefore, aging should rather be considered a modifier of mitochondrial dysfunction and metabolic vulnerability in T2DM, rather than an independent mitophagy-mediated disorder. Further research is needed to clarify the impact of mtDNA heteroplasmy and mitophagy on aging and their association with T2DM.

## 7. The Role of Mitophagy in Cardiovascular Diseases Associated with T2DM

Mitochondrial integrity is vital for optimal cardiac function. Defects in mitochondrial dynamics are known to contribute to cardiovascular conditions such as ischemic heart disease, heart failure, and atherosclerosis [117]. In the context of T2DM, hyperglycemia, altered lipid metabolism, oxidative stress, and inflammation place additional metabolic pressure on cardiomyocytes. One study reported impaired mitochondrial respiration and dynamics, together with reduced myocardial contractile function in T2DM patients. This study did not directly measure mitophagic flux and therefore did not provide any proof of defective mitophagy [118].

Genetic studies in mice have shown that proteins controlling mitochondrial fission, fusion, and selective mitochondrial removal are required for cardiac homeostasis. Among these proteins, Drp1 regulates mitochondrial fission; Mfn1 and Mfn2 regulate fusion; FOXO3 is a transcription factor; and PINK1, Parkin, and BNIP3 participate more directly in mitophagy [119]. Mitophagy acts as both a protective and a damaging mechanism for cardiac function, depending on its extent and on the nature and duration of cardiac stress. Drp1 plays a critical role in mitophagy, partly by separating damaged mitochondrial segments before their removal. In mouse hearts, Drp1-dependent mitochondrial autophagy protects against energy stress and pressure-overload-induced mitochondrial dysfunction [120,121]. Uncontrolled mitophagy resulting from Drp1 loss can lead to lethal cardiomyopathy [122,123]. Interestingly, Drp1 inhibition has shown beneficial effects in experimental pulmonary arterial hypertension; however, these findings were obtained outside the context of diabetes and primarily concern mitochondrial fission rather than mitophagy [124,125,126]. These findings illustrate the context-dependent role of mitochondrial fission and should not be directly extrapolated to T2DM [126].

Regarding diabetic cardiomyopathy, most of the evidence linking defective mitophagy to myocardial damage derives from cellular and animal studies. Using an STZ-induced β-cell injury/insulin-deficient diabetic mouse model, researchers demonstrated that Sirtuin-3 deficiency exacerbated cardiac dysfunction, mitochondrial damage, fibrotic remodeling, and cardiomyocyte apoptosis. Mechanistically, this was attributed to diminished FOXO3A deacetylation, decreased Parkin expression, and consequent inhibition of mitophagy [127]. It is important to emphasize that these findings are only preclinical evidence of diabetic cardiac injury.

Other experimental models indicate that more than one mitophagy pathway operates in the metabolically stressed heart. In mice exposed to a high-fat diet, ULK1-Rab9-dependent alternative mitophagy was activated during prolonged obesity and limited the development of obesity-associated cardiomyopathy [128]. In an HFD/STZ model incorporating insulin resistance and β-cell dysfunction, ZIP7 upregulation suppressed PINK1/Parkin-dependent mitophagy, whereas cardiac ZIP7 deletion preserved mitophagy and reduced fibrosis and cardiac dysfunction [129]. Most evidence linking dysregulated mitophagy to myocardial injury in diabetes derives from cellular and animal studies and should therefore be interpreted as preclinical evidence. In adult male wild-type and Sirt3-knockout mice, STZ treatment for 3 months resulted in cardiac dysfunction, interstitial fibrosis, cardiomyocyte apoptosis, mitochondrial injury, and suppression of autophagy and mitophagy. Importantly, this STZ-alone model primarily produces pancreatic β-cell injury, insulin deficiency, and hyperglycemia and should not be considered a direct experimental equivalent of human T2DM. Taken together, these findings suggest that mitophagy is dynamically regulated across different experimental models of diabetes and metabolic stress; however, the specific pathways involved may depend on the metabolic context and experimental model. Because these findings are derived predominantly from preclinical models, their relevance to human diabetic cardiomyopathy remains to be established.

Collectively, these findings support a protective role for mitochondrial quality control in experimental diabetic cardiomyopathy, while indicating that the predominant pathway involved is context-dependent, varying with the disease model and stage of progression. Data derived from human cardiac tissue remain scarce, however, and none specifically addresses diabetic cardiomyopathy. In one report, PINK1 protein levels were found to be decreased in myocardial specimens obtained from patients with end-stage heart failure, and mice lacking PINK1 exhibited mitochondrial dysfunction, increased oxidative stress, cardiac hypertrophy, fibrosis, and impaired left ventricular function [130]. Although this work underscores a role for PINK1 in maintaining cardiac homeostasis, it does not demonstrate that PINK1 deficiency causally contributes to diabetic cardiomyopathy in humans.

Mitophagy also modulates myocardial ischemia–reperfusion injury, a process of particular clinical significance given the elevated prevalence of ischemic cardiovascular disease among individuals with T2DM. During the ischemic and reperfusion phases, mitochondrial membrane depolarization, intracellular calcium overload, excessive ROS generation, and opening of the mitochondrial permeability transition pore collectively contribute to cardiomyocyte injury. Prompt clearance of irreversibly damaged mitochondria may mitigate this damage; conversely, mitophagy that is excessive or sustained beyond an optimal window may deplete the pool of functional mitochondria, thereby impairing myocardial recovery. Accordingly, the net impact of mitophagy on IRI appears to be governed by its temporal dynamics, magnitude, and the specific molecular pathway engaged. Beyond cardiovascular complications, mitophagy is also implicated in diseases that share metabolic and inflammatory features with T2DM, including cancer.

## 8. The Role of Mitophagy in Cancer and Its Relevance to T2DM

Mitophagy has context-dependent roles in cancer. In non-transformed cells, removal of damaged mitochondria may limit ROS-associated cellular injury, whereas established tumors can exploit mitophagy to survive metabolic, oxidative, and hypoxic stress [131,132]. Thus, the consequences of mitophagy depend on the biological and disease context.

Cancer is relevant to T2DM because the two conditions share metabolic features, including obesity, hyperinsulinemia, chronic inflammation, altered nutrient signaling, and oxidative stress [133,134]. However, direct evidence that altered mitophagy mechanistically links T2DM with cancer in humans remains limited [133].

Experimental cancer studies further demonstrate the context-dependent functions of PINK1/Parkin-mediated mitochondrial quality control; however, these findings should not be directly extrapolated to T2DM [135,136,137,138,139,140,141,142,143]. PINK1/Parkin signaling is also involved in mitochondrial quality control in pancreatic β-cells and insulin-responsive tissues [68,144,145]. In diabetes, impaired mitochondrial clearance may contribute to dysfunctional mitochondrial accumulation, increased ROS, impaired glucose-stimulated insulin secretion, and IR. Therefore, mitochondrial quality-control pathways may represent a biological point of convergence between metabolic stress in T2DM and cancer, although direct human evidence remains limited.

Several anticancer agents can modulate mitophagy in experimental models. Sorafenib promotes Parkin recruitment following mitochondrial electron-transport-chain disruption, whereas ketoconazole induces excessive mitophagy and apoptosis in hepatocellular carcinoma models [146,147]. However, these findings remain preclinical and do not establish these agents as validated mitophagy-directed therapies, particularly in patients with T2DM. Overall, cancer studies highlight the context-dependent role of mitophagy, while direct translational relevance to T2DM remains limited. Obesity and metabolic syndrome have a more direct pathophysiological relationship with T2DM.

## 9. The Role of Mitophagy in Obesity, Metabolic Syndrome and Its Relevance to T2DM

Metabolic syndrome (MetS) comprises a cluster of interrelated metabolic dysregulations, including central obesity, elevated fasting glucose, hypertriglyceridemia, reduced high-density lipoprotein cholesterol, and elevated blood pressure [148]. These disturbances substantially increase the risk of developing T2DM because they promote systemic IR and progressively increase the demand placed on pancreatic β-cells [149,150]. Physical inactivity and prolonged consumption of an energy-dense diet are among the major modifiable contributors, although genetic susceptibility, aging, sleep disruption, medications, and socioeconomic and environmental factors also influence disease development [151,152].

In obesity and MetS, chronic nutrient excess alters mitochondrial substrate oxidation, biogenesis, fission–fusion balance, and redox homeostasis in the liver, skeletal muscle, adipose tissue, and pancreatic β-cells [27,153,154,155]. Accumulation of lipid intermediates and uncontrolled ROS impair insulin signaling, promote inflammation, and reduce metabolic flexibility [156,157,158]. In skeletal muscles, these changes impair glucose uptake and oxidation. In the liver, they enhance lipid accumulation and gluconeogenesis. In adipose tissues, they promote inflammatory cytokine release and increase free-fatty-acid flux. Collectively, these tissue-specific abnormalities aggravate IR and increase β-cell workload, thereby facilitating the progression from MetS to T2DM. However, most human studies remain observational, and mitochondrial dysfunction may be both a cause and a consequence of obesity, physical inactivity, hyperglycemia, or lipid overload [159,160].

Mitophagy is increasingly recognized as a critical factor in the transition from obesity and MetS to T2DM [161]. In metabolically stressed tissues, insufficient mitochondrial clearance can allow dysfunctional mitochondria to accumulate, leading to persistent ROS production, defective ATP generation, impaired insulin signaling, and inflammation. Excessive or poorly coordinated mitophagy can deplete mitochondrial mass and compromise energy production in tissues with high metabolic demands. Thus, T2DM is more accurately associated with tissue-specific dysregulation of mitophagic flux than with uniform suppression of mitophagy.

This concept is particularly relevant to metabolic dysfunction-associated steatotic liver disease (MASLD; formerly termed non-alcoholic fatty liver disease, or NAFLD). The updated term MASLD better reflects the close relationship between hepatic steatosis and metabolic risk factors such as obesity, IR, and T2DM [161,162]. In the liver, defective mitochondrial quality control contributes to lipid accumulation, oxidative stress, and impaired insulin action, thereby reinforcing hepatic IR and hyperglycemia [163]. Prolonged high-fat diet (HFD) consumption has been associated with altered mitophagy in several experimental models, but the direction of this change is not uniform across tissues and disease stages [164,165]. Most direct mechanistic evidence still comes from cultured cells and rodent models rather than human metabolic tissues [163,164].

The inhibition of mitophagy exacerbates obesity and triglyceride accumulation [165]. The tissue-specific effects of FUNDC1 illustrate this complexity. In mice with skeletal-muscle-specific deletion of *Fundc1*, mitophagy and mitochondrial energetics were impaired. Unexpectedly, these mice exhibited partial protection from diet-induced obesity along with improved insulin sensitivity and glucose tolerance. The proposed mechanism involved mitochondrial stress-induced secretion of fibroblast growth factor 21 (FGF21), which promoted thermogenic remodeling of adipose tissue [163]. This finding does not indicate that inhibiting mitophagy is generally beneficial in T2DM; rather, it suggests that compensatory muscle–adipose endocrine signaling can transiently offset local mitochondrial dysfunction. In contrast, *Fundc1* deficiency in another model worsened HFD-induced obesity and IR. Loss of FUNDC1 impaired mitochondrial quality control in white adipose tissue and was associated with adipocyte dysfunction, macrophage infiltration, and increased inflammation [166]. These changes are directly relevant to T2DM because inflamed adipose tissue releases excess fatty acids and cytokines that worsen hepatic and skeletal-muscle IR and increase β-cell stress. The opposing findings across tissues suggest that the metabolic consequences of FUNDC1 loss depend on the affected organ and on compensatory communication among skeletal muscle, adipose tissue, liver, and pancreas [166]. They also suggest that systemic activation or inhibition of a single mitophagy pathway could produce unintended effects in patients with T2DM.

ULK1-dependent mitophagy influences the progression from obesity to T2DM. The nuclear receptor NR1D1 has been reported to promote ULK1-dependent mitophagy in adipocytes. In preclinical adipocyte models, NR1D1 upregulation enhanced LC3 expression and reduced mitochondrial ROS, triglyceride accumulation, and lipid-droplet formation, whereas ULK1 knockdown or pharmacological inhibition of mitophagy weakened these effects [165,166]. Targeting the NR1D1–ULK1 axis may make it possible to enhance mitochondrial quality in adipocytes and limit the spillover of lipids and inflammatory mediators, thereby attenuating systemic IR and reducing the risk of progression to T2DM. Nevertheless, this mechanism has yet to be confirmed in clinical populations, and questions regarding safety, tissue specificity, and therapeutic efficacy remain open.

Taken together, obesity and MetS provide a mechanistic framework linking impaired mitophagy to the development of T2DM. Dysregulation of mitochondrial turnover across adipose tissue, skeletal muscle, liver, and pancreatic β-cells appears to contribute to IR, ectopic lipid deposition, inflammation, and β-cell dysfunction. Nonetheless, the current evidence supports a tissue- and context-dependent model, indicating that therapeutic approaches should focus on restoring proper mitophagic flux rather than uniformly promoting or suppressing mitochondrial clearance. The tissue-specific nature of these mitophagic responses also highlights the importance of understanding how mitochondrial quality-control pathways are regulated at the gene-expression level. ncRNAs represent one such regulatory mechanism and are discussed below.

## 10. Noncoding RNAs Dysfunction in Diabetes

Mitochondria are both an important source and a major target of intracellular ROS. In T2DM, chronic hyperglycemia, lipotoxicity, and inflammation can increase mitochondrial ROS production in pancreatic β-cells and insulin-responsive tissues. High levels of free radicals induce mutations in mitochondrial DNA (mtDNA) and lead to damaged/dysfunctional mitochondria [167]. Oxidative stress can also damage mitochondrial proteins and membrane lipids, further impairing mitochondrial respiration and quality-control mechanisms. These changes may compromise ATP-dependent insulin secretion in β-cells and contribute to IR in the liver, skeletal muscle, and adipose tissue. Other alterations affecting mtDNA integrity (e.g., copy-number depletion, heteroplasmy, epigenetic modifications), the mtDNA-encoded transcriptome, or ncRNAs have also been linked to mitochondrial dysfunction [22,168]. The relationship between mitochondrial dysfunction and ncRNAs is bidirectional: diabetes-associated metabolic and oxidative stress can alter ncRNA expression, while ncRNAs can regulate nuclear- and mitochondrial-encoded genes involved in mitochondrial biogenesis, dynamics, respiration, and degradation.

ncRNAs are a diverse class of regulatory transcripts that generally do not encode proteins but perform important transcriptional, post-transcriptional, and epigenetic functions [169,170]. They include small ncRNAs, such as microRNAs, snoRNAs, and snRNAs, typically under 200 nucleotides in length; lncRNAs, and circRNAs derived from linear or back-spliced transcripts [171,172]. In T2DM, these RNA classes participate in gene regulatory networks governing glucose-stimulated insulin secretion, β-cell survival, insulin sensitivity, glucose and lipid metabolism, inflammation, and cellular stress responses. miRNAs, lncRNAs, and circRNAs often act within interconnected networks, functioning as sponges, decoys, scaffolds, or guides for protein and RNA complexes [19]. Depending on their cellular localization and binding partners, lncRNAs can function as scaffolds, guides, decoys, or competing endogenous RNAs. Many circRNAs are relatively resistant to exonuclease-mediated degradation because they lack free 5′ and 3′ ends. Some circRNAs can bind miRNAs or proteins, although miRNA sponging should not be considered a universal function of all circRNAs [173].

The biogenesis and regulation of ncRNAs involve both canonical transcriptional machinery and non-canonical processing events [174]. For instance, miRNAs are transcribed by RNA polymerase II, processed by Drosha/DGCR8 into pre-miRNAs, and subsequently by Dicer into mature miRNAs [175,176]. Many lncRNAs are also transcribed by RNA polymerase II and undergo capping, splicing, and polyadenylation, although their processing and subcellular localization vary considerably [177]. CircRNAs, in contrast, are formed by back-splicing of exons or introns, often facilitated by complementary intronic sequences or RNA-binding proteins [178]. Tissue specificity is particularly important in T2DM, because an ncRNA detected in blood may not accurately reflect its expression or biological function in pancreatic islets, liver, skeletal muscle, adipose tissue, kidney, heart, retina, or vascular endothelium [20]. Consequently, circulating ncRNAs should not be considered reliable diabetes biomarkers without validation against tissue-specific disease processes and independent patient cohorts.

Functionally, ncRNAs contribute to several key biological processes relevant to diabetes, including the control of pancreatic β-cell differentiation and survival; the refinement of insulin-signaling cascades in peripheral tissues; the regulation of glucose and lipid metabolism; the coordination of inflammatory, oxidative-stress and ERS responses; and the modulation of vascular, renal, and neural cellular functions [179,180]. Aberrant lncRNA expression has been directly associated with endothelial dysfunction, vascular smooth muscle cell phenotypic changes, and macrophage inflammatory activation in diabetic peripheral artery disease [181]. Their principal relevance to this review lies in their ability to regulate mitochondrial quality control and thereby influence β-cell failure, IR, and diabetic complications. Selected ncRNAs have been reported to influence PINK1/Parkin-dependent mitophagy, DRP1-mediated mitochondrial fission, and signaling pathways involving AMPK, mTOR, ULK1, sirtuins, and BNIP3/NIX. Through these mechanisms, ncRNAs may determine whether damaged mitochondria are repaired, retained, or removed during glucotoxic, lipotoxic, and inflammatory stress in T2DM.

Direct examples have been reported in experimental models of diabetic kidney disease. In high-glucose-treated renal tubular epithelial cells, lncRNA NEAT1 promoted cellular injury by regulating the miR-150-5p-DRP1 axis and altering mitophagy [182]. In podocyte and diabetic nephropathy models, knockdown of lncRNA SNHG17 promoted Parkin-dependent mitophagy through regulation of MST1 and reduced podocyte apoptosis [183]. These studies directly link ncRNA-mediated control of mitochondrial turnover to renal-cell injury in diabetes. However, the evidence derives mainly from cultured cells and animal models and requires validation in human diabetic kidney tissue.

Collectively, these findings indicate that ncRNAs either promote or restrain mitophagy depending on the target, tissue, and stage of diabetic injury. Therefore, increased mitophagy should not necessarily be interpreted as beneficial. In T2DM, the therapeutic goal would be to restore optimal mitophagic flux in specific tissues rather than to indiscriminately induce mitochondrial clearance. Evidence from obesity-related cardiac models provides mechanistic insight into metabolic stress but should be distinguished from studies performed in clinically confirmed diabetic cardiomyopathy [184].

## 11. Functional Roles and Translational Relevance of ncRNAs in T2DM

**Figure 3:**
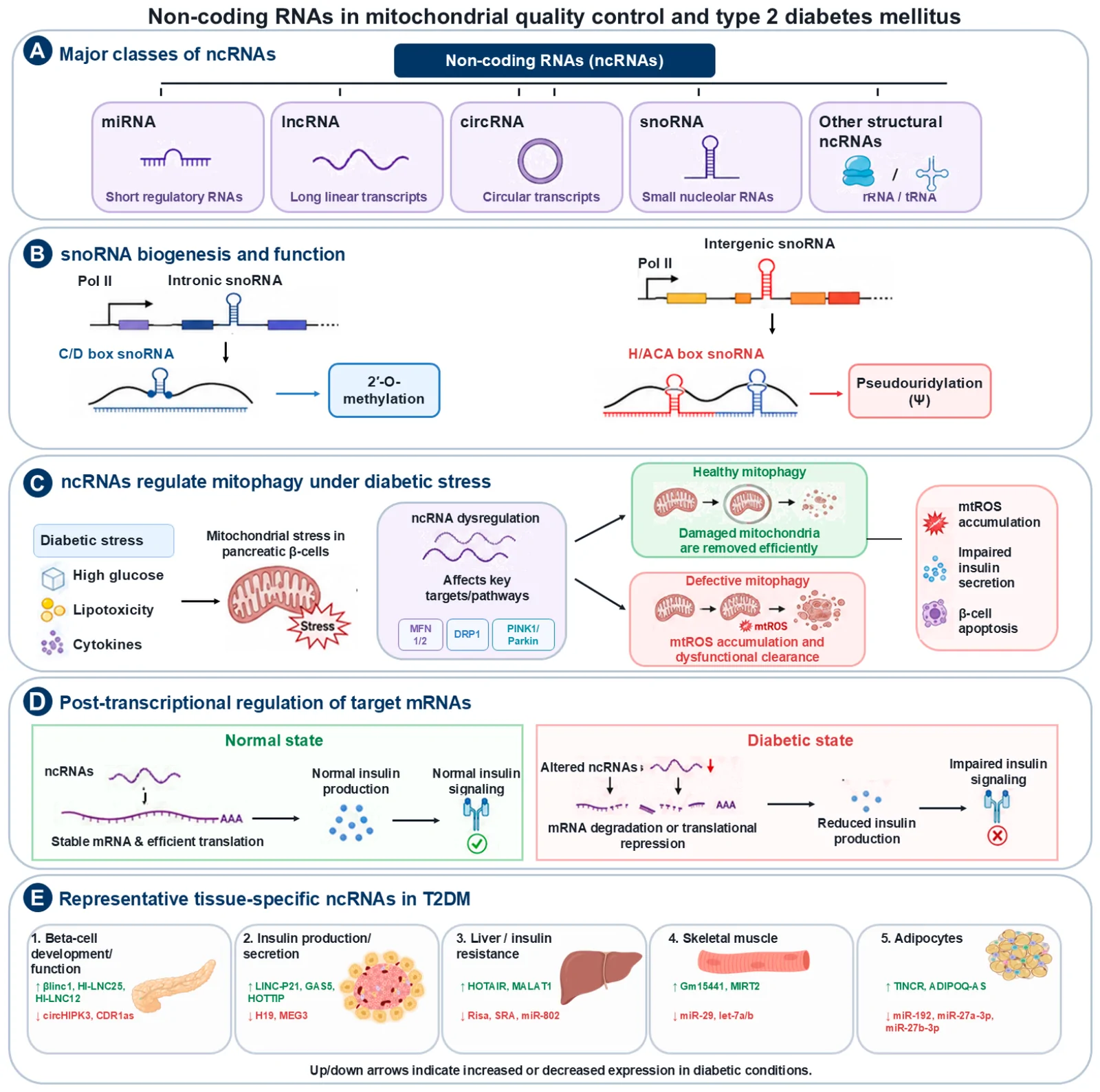
Noncoding RNAs in mitochondrial quality control and type 2 diabetes mellitus; classification, biogenesis and tissue-specific roles. (**A**) The ncRNAs are structurally and functionally diverse. They are commonly classified according to their genomic origin, transcript length, and molecular configuration. These classes include cis-regulatory RNAs, transfer and ribosomal RNAs, small nuclear and small nucleolar RNAs, intron-derived transcripts, long noncoding RNAs, antisense RNAs, and circular RNAs. (**B**) Small nucleolar RNAs (snoRNAs) can originate from intronic or intergenic regions and are mostly transcribed by RNA polymerase II. C/D-box snoRNAs primarily guide 2′-O-methylation, whereas H/ACA-box snoRNAs guide pseudouridylation of target RNAs. (**C**) Dysregulated ncRNAs can modulate mitochondrial fission and PINK1/Parkin-dependent mitophagy by targeting MFN proteins, DRP1, and PINK1 under diabetic metabolic stress conditions such as hyperglycemia, lipotoxicity, oxidative stress, and inflammatory cytokine exposure. Impaired clearance of damaged mitochondria leads to accumulation of mitochondrial ROS, defective mitophagy, impaired insulin secretion, and pancreatic β-cell apoptosis. (**D**) Under physiological conditions, ncRNAs modulate target mRNA stability and translation, fostering β-cell development and insulin production. In diabetes, altered ncRNA expression may contribute to β-cell dysfunction and IR by promoting transcript degradation or suppressing transcripts involved in insulin biosynthesis and signaling. (**E**) Representative ncRNAs show tissue- and context-specific expression patterns in pancreatic β-cells, liver, skeletal muscle, and adipose tissue, regulating insulin production, glucose metabolism, mitochondrial homeostasis, and insulin sensitivity. Up and down arrows indicate increased and decreased expression, respectively, under diabetic conditions. Collectively, the figure illustrates how different classes of ncRNAs integrate epigenetic, post-transcriptional, and mitochondrial quality-control mechanisms in the pathophysiology of type 2 diabetes mellitus (T2DM).

Noncoding RNAs influence several biological processes relevant to T2DM, including pancreatic β-cell development and insulin secretion, insulin signaling in peripheral tissues, mitochondrial quality control, inflammation, and the development of diabetic complications. Their effects are highly tissue- and context-dependent, and the same ncRNA can have different consequences in pancreatic islets, liver, skeletal muscle, adipose tissue, kidney, heart, or vascular cells. Figure 3 summarizes the major classes and regulatory mechanisms of ncRNAs and presents representative ncRNAs associated with pancreatic β-cell function, IR, skeletal muscle, and adipose tissue. The figure highlights ncRNAs directly involved in mitochondrial quality control, insulin secretion, insulin sensitivity, or diabetic complications. The evidence supporting these associations ranges from cell-based experiments and animal models to human tissues and circulating samples. Accordingly, mechanistic findings should be distinguished from observational biomarker associations and from clinically validated applications.

### 11.1. ncRNAs in Pancreatic β-Cell Identity and Insulin Secretion

miRNAs are central modulators of gene expression at the post-transcriptional level, and in diabetes, their dysregulation is both a cause and a consequence of metabolic dysfunction. Under normal physiological conditions, miRNAs help maintain β-cell homeostasis, regulate insulin biosynthesis and secretion, and modulate downstream insulin-signaling pathways in peripheral tissues, including skeletal muscle, adipose tissue, and liver. In T2DM, chronic glucotoxicity, lipotoxicity, oxidative stress, and ER stress can alter β-cell miRNA expression, potentially impairing mitochondrial ATP production, insulin granule exocytosis, and β-cell survival.

Global profiling studies have identified miRNAs that are differentially expressed in prediabetes, T2DM, and insulin-resistant states, including miRNAs associated with the PI3K-AKT, JAK-STAT, and endoplasmic reticulum (ER) stress pathways. However, differential expression alone does not demonstrate that a given ncRNA causes β-cell failure or IR. Much of the current evidence comes from cross-sectional profiling studies, cultured cells, and rodent models. The tissue source, disease stage, metabolic treatment, and method used to measure ncRNA expression should therefore be considered when interpreting these findings. Blood-cell composition, hemolysis, renal function, obesity, and antidiabetic medications also affect circulating miRNA levels, meaning these levels do not necessarily reflect ncRNA expression within pancreatic islets.

Several lncRNAs have more direct experimental support for roles in β-cell development and function. In mice, deletion of βlinc1 impaired islet development, altered the expression of neighboring β-cell transcriptional regulators, and disrupted glucose homeostasis. This finding supports a functional role for βlinc1 but does not establish it as a therapeutic target in humans [185]. Such findings are relevant to T2DM because impaired maintenance of β-cell identity and function can reduce compensatory insulin secretion during IR and accelerate progression from prediabetes to overt diabetes. However, confirmation in human islets and longitudinal patient cohorts remains limited.

ncRNAs can also influence β-cell function by regulating mitochondrial dynamics and mitophagy, directly targeting specific mitochondrial quality-control pathways. For instance, lncRNA NEAT1 modulates the miR-150-5p–DRP1 axis, altering mitochondrial fission and mitophagy in renal tubular epithelial cells exposed to high glucose [182]. In experimental podocyte models, lncRNA SNHG17 inhibits Parkin-dependent mitophagy via MST1 [183]. LncRNA H19 modulates PINK1/Parkin-mediated mitophagy and mitochondrial respiration in obesity-induced cardiac dysfunction [184]. Other ncRNAs affect broader autophagy-related pathways, such as SIRT1/GSK3β signaling, but should not be cited as direct evidence of mitophagy unless selective mitochondrial clearance has been specifically demonstrated [185]. These context-dependent mechanisms may drive ncRNA-induced alterations in mitochondrial turnover, respiration, ROS accumulation, and apoptotic susceptibility. Collectively, these results demonstrate pathway-specific associations between ncRNA dysregulation and mitochondrial quality control in diabetic and metabolic-stress models. However, their direct relevance to pancreatic β-cell failure in T2DM is not yet fully established, and only a fraction of the proposed ncRNA–target interactions has been validated by mechanistic assays.

Dysregulated miRNAs (oncogenic or tumor-suppressor miRNAs), lncRNAs (such as those modulating chromatin modifiers or transcription-factor networks), and circRNAs have also been implicated in pancreatic ductal adenocarcinoma, islet cell tumors, and the pre-neoplastic transformation of β-cells under chronic IR or hyperinsulinemia [186,187,188]. However, because this review focuses on T2DM, these cancer-related observations are summarized only briefly, as an illustration of the consequences of long-standing hyperinsulinemia, oxidative stress, and inflammatory signaling. These ncRNAs alter cell-cycle control, apoptosis resistance, angiogenesis, and interactions with the tumor microenvironment [189]. Moreover, the chronic hyperglycemia, oxidative stress, and inflammation observed in diabetes have been reported to disrupt ncRNA expression, thereby facilitating epigenetic reprogramming and oncogenic signaling [190].

These mechanisms point to a plausible biological convergence between T2DM and pancreatic tumorigenesis; however, direct human evidence linking ncRNA-mediated mitophagy to both conditions is currently lacking. Consequently, ncRNAs cannot yet be regarded as clinically validated biomarkers or therapeutic targets for diabetes-associated pancreatic cancer. While experimental modulation of specific miRNAs or lncRNAs has been shown to reduce tumor growth in murine models, such findings demonstrate mechanistic plausibility rather than clinical efficacy or safety.

Overall, the principal contribution of the ncRNAs discussed in this section lies in their capacity to regulate β-cell identity, mitochondrial quality control, insulin secretion, and survival under metabolic stress. Translating these findings into clinical applications for T2DM will require validation in human islets, standardized methods for quantifying circulating ncRNAs, replication across independent cohorts, and evidence that targeted modulation improves β-cell function without inducing off-target effects.

### 11.2. ncRNA-Mediated Regulation of Mitophagy and Mitochondrial Quality Control

Among the ncRNAs relevant to the central theme of this review, those that directly regulate mitochondrial quality-control pathways are of particular interest. Experimental studies suggest that ncRNAs can affect mitochondrial fission and selective mitophagy, for example, through the miR-150-5p–DRP1 axis, MST1-Parkin signaling, and PINK1/Parkin-dependent mitochondrial clearance [182,183,184]. In T2DM, the chronic hyperglycemia, lipotoxicity, oxidative stress, and inflammation cause mitochondrial dysfunction and cellular injury in pancreatic β-cells and insulin-sensitive tissues [191,192,193,194]. This suggests that dysregulation of ncRNA-mediated mitochondrial turnover may contribute to defective insulin secretion, IR, and the development of diabetic complications. However, this mechanistic association is tissue- and model-specific and has not been uniformly validated in human T2DM tissues.

The biological consequences of ncRNA-mediated mitophagy regulation vary depending on tissue type and cellular context. Certain ncRNAs impair the removal of damaged mitochondria, while others limit excessive mitophagy. For example, in renal tubular epithelial cells exposed to high-glucose conditions, the lncRNA NEAT1 was shown to exacerbate cellular damage by modulating the miR-150-5p–DRP1 axis, thereby influencing mitochondrial fission and mitophagy [182]. This mechanism is particularly relevant to diabetic kidney disease, as excessive mitochondrial fragmentation combined with impaired mitochondrial turnover can drive increased ROS production, tubular cell damage, and renal inflammation under sustained hyperglycemia. Nevertheless, current evidence derives largely from in vitro studies, and validation in human diabetic kidney tissue is still needed.

In podocyte models, increased lncRNA SNHG17 expression suppressed Parkin-dependent mitophagy by regulating the kinase MST1, whereas SNHG17 knockdown restored mitochondrial clearance and reduced podocyte apoptosis. These findings suggest that the SNHG17-MST1-Parkin axis may link hyperglycemia-induced ncRNA dysregulation to podocyte mitochondrial injury and albuminuria in T2DM. Nevertheless, this pathway has not yet been validated in human kidney tissue or prospectively linked to diabetic nephropathy progression [183].

The effects of lncRNA H19 illustrate why mitophagy should not always be increased without considering the biological context. In experimental obesity-associated cardiac dysfunction, H19 limited PINK1 mRNA translation and reduced excessive PINK1/Parkin-mediated mitophagy, thereby improving mitochondrial respiration. Obesity and IR are major risk factors for T2DM and diabetic cardiomyopathy; therefore, this model offers mechanistic insight into how excessive mitochondrial clearance may contribute to cardiac energetic failure under metabolic stress. However, it does not establish the same mechanism in clinically confirmed T2DM or diabetic cardiomyopathy patients [184].

High expression of Risa is observed in diabetic nephropathy, where it aggravates podocyte injury by inhibiting autophagy through the SIRT1/GSK3β signaling pathway. Because this study examined general autophagy rather than selective mitochondrial autophagy, Risa should be discussed as an autophagy-regulating lncRNA rather than cited as direct evidence of mitophagy regulation [195]. Its relevance to T2DM lies in the broader role of SIRT1-dependent stress adaptation and autophagic homeostasis in protecting podocytes against glucotoxic and oxidative injury.

These examples demonstrate that the relationship between ncRNAs and mitochondrial quality control is not uniform. The same pathway may be beneficial when damaged mitochondria accumulate but harmful when mitochondrial removal exceeds the capacity for mitochondrial biogenesis. Therefore, in T2DM, the therapeutic goal should be to restore appropriate mitophagic flux in the affected tissue rather than indiscriminately activating or inhibiting mitophagy. Collectively, these findings show that ncRNAs regulate mitochondrial quality control through multiple tissue- and context-dependent mechanisms. Specific miRNAs, lncRNAs, circRNAs, and other small ncRNA classes may influence PINK1/Parkin-dependent mitophagy, mitochondrial fission, autophagy-mitophagy crosstalk, mitochondrial biogenesis, oxidative stress, and cellular survival. Representative ncRNAs implicated in mitophagy and mitochondrial quality control in T2DM and related metabolic diseases are summarized in Table 3.

### 11.3. Tissue-Specific Roles of ncRNAs in Diabetic Complications

The effects of ncRNAs vary considerably across the tissues involved in T2DM, including pancreatic β-cells, kidney, heart, vascular tissue, liver, skeletal muscle, adipose tissue, retina, and peripheral nerves. These tissues contribute to T2DM pathogenesis through distinct mechanisms: pancreatic β-cells govern the capacity for compensatory insulin secretion, whereas the liver, skeletal muscle, and adipose tissue are the principal determinants of systemic insulin sensitivity. Chronic diabetic complications, by contrast, primarily affect the kidney, heart, retina, peripheral nerves, and vascular tissue. Given these distinct pathophysiological roles, ncRNAs implicated in different diabetic complications should not be grouped into a single, undifferentiated list.

In diabetic kidney disease, ncRNAs have been implicated in podocyte loss, tubular injury, inflammation, fibrosis, autophagy, and mitochondrial dysfunction. For instance, the long noncoding RNA (lncRNA) MIAT was found to be upregulated in both plasma and kidney tissue of patients with diabetic nephropathy. Genetic and cell-based experiments demonstrated that MIAT contributes to podocyte injury via the miR-130b-3p-SOX4 axis [213]. This study integrated evidence from human samples with mouse and cellular models; however, the mechanistic pathway identified was unrelated to mitophagy. MIAT should therefore be considered a regulator of diabetic kidney injury, but not a direct mitophagy-related lncRNA.

miR-130b has been investigated as a potential biomarker for diabetic nephropathy. A study of human serum reported an association between circulating miR-130b levels and the severity of albuminuria. However, this is observational biomarker evidence and does not establish that circulating miR-130b reflects its activity within renal cells, nor that it drives disease progression [214]. Confirming its clinical value in T2DM will require external validation, longitudinal follow-up, standardized measurement protocols, and adjustment for renal function, medication use, obesity, and other potential confounders.

H19, MEG3, MALAT1, and HOTAIR have likewise been implicated in inflammation, fibrosis, IR, and diabetes-associated vascular, retinal, renal, and cardiac damage. Nonetheless, the expression patterns of these ncRNAs are often inconsistent across tissues, diabetes subtypes, and experimental settings. An ncRNA that exerts a protective effect in pancreatic β-cells, for instance, may instead promote fibrotic or inflammatory processes within the kidney or vasculature. Accordingly, rather than being characterized simply as globally up- or downregulated biomarkers, these molecules should be classified according to tissue specificity, molecular target, associated diabetic phenotype, and level of supporting evidence.

Organizing the discussion by tissue can clearly establish the relevance of these ncRNAs to T2DM pathophysiology. Specifically, ncRNAs influencing β-cell mitochondrial function should be interpreted in relation to insulin secretion and β-cell survival; those active in liver and skeletal muscle should be discussed with respect to glucose production, substrate oxidation, and insulin sensitivity; adipose tissue-associated ncRNAs should be examined in terms of lipolysis and inflammatory signaling; and ncRNAs identified in renal, cardiac, retinal, neural, and vascular tissues should be considered within the context of their corresponding diabetic complications.

### 11.4. Circulating ncRNAs as Candidate Biomarkers

Circulating miRNA signatures are being explored clinically as early biomarkers of diabetes onset or progression, reflecting tissue- and cell-type-specific changes in miRNA release [215]. They may be useful for detecting molecular changes before the development of irreversible β-cell failure or advanced organ damage. However, circulating ncRNA abundance does not necessarily indicate tissue of origin or establish a mechanistic role in T2DM. The main challenge lies in translating an observed ncRNA signature into a reproducible and clinically informative marker. Hemolysis, blood cell composition, sample processing time, choice of anticoagulant, storage conditions, renal clearance, obesity, inflammation, and antidiabetic treatment may all influence circulating ncRNA levels. Variations in RNA extraction, normalization, and analytical platforms further hinder cross-study comparisons.

Although not circulating biomarkers themselves, several tissue-derived ncRNAs provide mechanistic context for interpreting candidate circulating signatures. Animal studies have shown that knocking out the βlinc1 gene results in defective islet development and impaired glucose homeostasis, suggesting a potential role for targeted therapies aimed at restoring β-cell function [216]. HI-LNC25 depletion leads to downregulation of GLIS3 mRNA, which is crucial for normal β-cell function [216]. Pancreatic islet lncRNAs (HI-LNC12, HI-LNC25, HI-LNC75, HI-LNC78, and HI-LNC45) are also important, as they regulate β-cell maturation [217,218]. These findings are relevant to T2DM, since loss of β-cell identity and functional maturity may limit compensatory insulin secretion during IR. However, their roles have been defined mainly in experimental systems and have not yet been validated as circulating biomarkers or therapeutic targets in patients with T2DM.

The miR-16 and miR-499 (especially miR-499-5p) families are involved in gene expression related to insulin signaling and glucose metabolism. Since some of the evidence cited pertains mainly to type 1 diabetes, these miRNAs should only be kept if there is direct evidence of T2DM, prediabetes, IR or T2DM-related complications. Otherwise, they should be excluded from this section to preserve the review’s focus. As such, they are potential therapeutic targets for type 1 diabetes mellitus [219].

Circular RNAs such as circRNA-HIPK3, circANKRD36, and CRD1 function as miRNA sponges, regulating the expression of genes involved in cell proliferation, apoptosis, insulin secretion, and inflammation. They also serve as non-invasive biomarkers for the diagnosis, prognosis, and monitoring of diabetes-related complications [220]. LINC-P21 (p3134) and GAS5 both act as sponges for several miRNAs that regulate IGF-1 and other signaling pathways involved in glucose metabolism. HOTTIP is implicated in diabetic nephropathy through involvement of the Wnt/β-catenin pathway and sponging of miR-455-3p [221,222,223]. miR-146a is a key regulator of inflammatory responses, while miR-130b is a potential biomarker for diabetic nephropathy [214,224]. HI-LNC71, HI-LNC78, and HI-LNC12 play regulatory roles in diabetic complications [225]. H19 contributes to renal fibrosis by acting as a molecular sponge that regulates downstream gene expression involved in inflammation, apoptosis, and fibrosis [226]. MEG3 and MALAT1 are differentially expressed (up- or down-regulated) in T1DM, GDM, or diabetic retinopathy and are involved in inflammation and IR, serving as potential biomarkers for these conditions [227].

The available evidence for clinically relevant ncRNAs in T2DM spans human observational studies, animal models, and mechanistic cell-based experiments. Some ncRNAs have been associated with β-cell dysfunction, impaired insulin signaling, endothelial injury, inflammation, diabetic nephropathy, and other complications, whereas others have been proposed as circulating candidate biomarkers or potential biomarkers. Nevertheless, evidence from experimental models should be distinguished from associations observed in human samples, and most candidates still require independent validation, standardized measurement, and prospective clinical evaluation, as summarized in Table 4.

### 11.5. Therapeutic Considerations

Experimental manipulations of ncRNAs through mimics, antisense oligonucleotides, small interfering RNAs, viral vectors, or CRISPR-based strategies have been shown to modulate metabolic or tissue-specific phenotypes in various cell and animal models. In principle, such approaches could restore β-cell function, improve insulin sensitivity, attenuate inflammatory injury, or reinstate mitochondrial quality control in tissues affected by T2DM. Nevertheless, clinical translation of these strategies remains limited. Key obstacles include targeted delivery to pancreatic islets and other metabolic tissues, rapid degradation in the circulation, unintended uptake by non-target organs, immune activation, dose-dependent toxicity, and off-target regulation of multiple mRNAs. These issues are especially relevant in T2DM, given that therapy would likely require chronic rather than short-term administration. Repeated dosing may lead to cumulative off-target effects, potentially heightening liver, kidney, hematological and immunotoxicity.

A further complication is that a given ncRNA may exert distinct or even opposing effects across different tissues. For example, modulating a ncRNA to promote mitophagy in the kidney could inadvertently affect mitochondrial turnover in the heart, liver, skeletal muscle, or pancreatic β-cells. Similarly, enhancing PINK1/Parkin-dependent mitophagy may benefit tissues with accumulated damaged mitochondria but harm tissues with high energy demands if it causes excessive mitochondrial depletion. Consequently, therapeutic conclusions should be confined to the specific experimental model in which an intervention was evaluated. Advancing ncRNA-based therapies for T2DM will require the development of tissue-selective delivery platforms, precise dose control, comprehensive long-term safety assessment, evaluation of mitophagic flux rather than static marker expression, and validation of efficacy in human tissues through well-designed clinical trials. At present, ncRNA-based modulation of mitophagy should be regarded as an experimental therapeutic avenue rather than a clinically established treatment for T2DM.

## 12. Conclusions, Limitations, and Future Perspectives

In T2DM, altered mitochondrial dynamics and defective mitophagy allow damaged mitochondria to persist, generating excessive ROS, triggering inflammation, and promoting IR across metabolic tissues. This mitochondrial dysfunction in T2DM, mostly attributable to dysregulation of the PINK1, Parkin, and BNIP3/NIX pathways, also contributes to diabetic complications such as nephropathy, retinopathy, neuropathy, and cardiovascular diseases. The influence of impaired mitophagy in T2DM depends on the disease progression, metabolic triggers, molecular pathways and tissue types involved. Recent research has identified ncRNAs (specifically microRNAs, long noncoding RNAs, and circular RNAs) as key regulators of mitochondrial dynamics and mitophagy. These ncRNAs modulate mitochondrial function through tissue- and context-dependent mechanisms, offering potential opportunities for the development of new biomarkers and targeted treatments.

This narrative review analyzed evidence from studies using cells, animal models, human tissues, and circulating samples to discuss the influence of mitochondrial dysfunction, mitophagy, and ncRNAs on T2DM and its complications. Most of the studies analyzed were preclinical investigations. The evidence available was highly heterogeneous and largely based on cell and animal studies. Comparisons were further complicated by differences in study design, patient populations, sample types, and methods used to assess ncRNAs and mitophagy. Further work is needed to establish their specificity, safety, reproducibility, and clinical utility. This will require integrating longitudinal patient cohorts, well-characterized human tissue samples, functional assays, and tissue-specific multi-omics strategies.

Key research priorities include clarifying how mitophagy shifts across different stages of T2DM progression, identifying which ncRNAs directly govern mitochondrial turnover in humans, and determining whether mitophagy can be selectively modulated in one tissue without compromising mitochondrial quality control in others. Ultimately, clinical trials will be needed to establish the safety and efficacy of any mitophagy-targeting compound or ncRNA-based therapeutic strategy. Emerging biomarkers and treatments must also remain cost-effective, reproducible, and broadly applicable across varied patient populations. Resolving these issues will help distinguish which elements of mitophagy and ncRNA biology hold genuine clinical potential from those that remain mechanistic hypotheses awaiting further validation.

## Figures and Tables

**Table 1:** Mitophagy and Mitochondrial Quality-Control Pathways in Metabolic Tissues.

Tissue	Mitophagy/Mitochondrial Defect	Mitochondrial Outcome	Metabolic Consequence
Pancreatic β-cells	Impaired Parkin-dependent or CLEC16A-regulated mitophagy	Abnormal mitochondria, ↓ respiration and ATP, ↑ oxidative stress	Impaired glucose-stimulated insulin secretion and β-cell dysfunction [67,68,69]
Liver	Reduced PARKIN-mediated hepatocyte mitophagy	Accumulation of dysfunctional mitochondria and hepatic lipids/steatosis	Hepatic IR and fatty-liver progression [70,71]
Skeletal muscle	Excessive mitochondrial fission and disturbed mitochondrial quality control	Mitochondrial fragmentation, oxidative stress, depolarization and ↓ ATP production	Reduced insulin-stimulated glucose uptake and muscle IR [72,73]
Adipose tissue	Dysregulated mitophagy and mitochondrial turnover	Mitochondrial loss/dysfunction, ROS–NF-κB activation and adipose inflammation	Altered fatty-acid metabolism and systemic/hepatic IR [33,74]
Heart	Insufficient or maladaptive ATG7/Parkin-dependent mitophagy during metabolic stress	Damaged mitochondria, oxidative stress, lipotoxicity and cardiomyocyte injury	Cardiac dysfunction and diabetic cardiomyopathy [75,76,77]

**Table 2:** Key Mitophagy-Related Signaling Pathways and Their Metabolic-Consequences in Diabetes.

Mitophagy/Regulatory Pathway	Key Regulators	Tissues with Supporting Evidence	Functional Consequence
PINK1/Parkin ubiquitin-dependent mitophagy	PINK1, PRKN/Parkin, phospho-ubiquitin, OPTN, NDP52, TBK1, LC3/GABARAP	Pancreatic β-cells, liver; possible/context-dependent contribution in skeletal muscle	Defective pathway causes accumulation of damaged mitochondria, mitochondrial dysfunction and increased ROS [68,70,95,96]
BNIP3/NIX receptor-mediated mitophagy	BNIP3, BNIP3L/NIX, LC3/GABARAP	Heart, liver and adipose tissue	Either insufficient or excessive activation can disturb mitochondrial clearance, promoting oxidative stress, metabolic dysfunction or cell death [58,97,98,99]
AMPK-mTORC1-ULK1 regulatory axis	AMPK, mTORC1, ULK1, ATG13, FIP200	Skeletal muscle, liver and heart; broader autophagy regulation in adipose tissue	Reduced AMPK activity or excessive mTORC1 activity suppresses ULK1-dependent autophagy initiation and may impair mitophagy [100,101,102,103]
Insulin–IRS-PI3K-AKT-FOXO signaling	Insulin receptor, IRS1/IRS2, PI3K, AKT, FOXO1/FOXO3, mTORC1	β-cells, skeletal muscle, liver and adipose tissue	Impaired signaling causes IR and can indirectly dysregulate autophagy, mitophagy and mitochondrial metabolism [104,105,106,107]
Wnt/β-catenin metabolic signaling	Wnt ligands, Frizzled, LRP5/6, GSK3β, β-catenin, TCF7L2	Primarily pancreatic β-cells and adipose tissue; cardiac effects are mostly related to remodeling	Regulates β-cell insulin secretion, survival and proliferation; its direct role in diabetic mitophagy remains insufficiently established [108,109,110,111]

**Table 3:** Noncoding RNAs’ impact on mitophagy in T2DM and related metabolic diseases.

Noncoding RNA	Noncoding RNA Type	Potential Experimental Model	Impact on Mitophagy	Role in T2DM
miR-34a	miRNA	Diabetic liver, heart and β-cell studies	Increases oxidative stress and mitochondrial dysfunction	Promotes IR and β-cell injury [179]
miR-27a	miRNA	Cell models (mechanistic); metabolically relevant	Suppresses PINK1 translation, inhibits Parkin recruitment and mitophagy	Potential negative regulator of mitochondrial quality control [196]
miR-27b	miRNA	Cell models	Reduces mitochondrial clearance by inhibiting PINK1	Candidate contributor to mitochondrial dysfunction in IR [196]
miR-181a	miRNA	HFD mouse; adipocyte studies	Inhibits mitophagy by suppressing Parkin	Impairs adipocyte mitochondrial homeostasis during obesity/T2DM [197]
miR-195	miRNA	Diabetic cardiomyopathy	Promotes mitochondrial damage and apoptosis	Contributes to diabetic cardiac dysfunction [198]
miR-30 family	miRNA	Diabetic heart	Regulates mitochondrial turnover	Protective when preserved [179]
miR-21	miRNA	Metabolic and mitochondrial studies	Context-dependent regulation of mitochondrial homeostasis	Modulates diabetic mitochondrial injury [199]
lncRNA H19	lncRNA	Obese mice; diabetic heart	Restrains excessive mitophagy, preserves mitochondrial respiration	Protective in obesity-associated metabolic dysfunction; relevant to T2DM cardiomyopathy [200]
lncRNA Blnc1	lncRNA	HFD mice; adipose tissue	Improves mitochondrial metabolism	Protects against obesity-associated IR [201]
MALAT1	lncRNA	Diabetic kidney and endothelial cells	Modulates oxidative stress and mitochondrial dysfunction	Important in diabetic complications [202,203]
MEG3	lncRNA	β-cell and diabetic models	Promotes mitochondrial dysfunction	Associated with β-cell injury and IR [204]
HOTAIR	lncRNA	Diabetic complication models	Regulates oxidative stress and mitochondrial integrity	Emerging MQC regulator [205]
circHIPK3	circRNA	Human islets; β-cell models	Maintains β-cell survival and insulin secretion	Protective in T2DM [206]
circRNA CDR1as	circRNA	β-cells	Improves insulin secretion and cell survival	Indirect regulator of mitochondrial homeostasis [207]
circ-PTPN4	circRNA	Skeletal muscle studies	Enhances mitochondrial activity	Potential relevance to insulin sensitivity [208]
Mitochondrial-derived lncRNAs (LIPCAR)	mito-lncRNA	Human metabolic and cardiovascular studies	Biomarker of mitochondrial dysfunction	Potential circulating biomarker in diabetes-associated cardiovascular disease [200,209]
tRNA-derived fragments (tRFs)	tRF	Human plasma and experimental models	Influence mitochondrial metabolism and stress responses	Early evidence; mechanisms still evolving [210]
piRNAs	piRNA	Limited metabolic studies	Putative mitochondrial regulatory role	Insufficient evidence in T2DM [211]
snoRNAs	snoRNA	Human metabolic studies	Associated with metabolic homeostasis	Limited mechanistic evidence [211,212]

**Table 4:** Clinically relevant noncoding RNAs in type 2 diabetes mellitus: human, animal, and cell studies.

ncRNA	ncRNA Class	Human Study	Animal Model	Cell Model	Expression in T2DM	Validated Target Gene(s)	Major Signaling Pathway	Major Biological Function	Clinical Significance
miR-375	miRNA	Serum and pancreatic islets from T2DM patients	STZ mice, db/db mice	INS-1, MIN6	↑	PDK1, MTPN	PI3K/Akt	Regulates insulin secretion and β-cell survival	Potential biomarker; β-cell dysfunction [228]
miR-126	miRNA	Plasma/serum of T2DM and prediabetes	Diabetic mice	Endothelial cells (HUVEC)	↓	IRS-1, VEGF pathway	PI3K/Akt/eNOS	Endothelial dysfunction and angiogenesis	Early biomarker of T2DM and vascular complications [229]
miR-29a	miRNA	Serum and skeletal muscle	HFD mice	C2C12 myotubes	↑	IRS-1, AKT2	Insulin signaling	Promotes IR	Candidate biomarker of IR [230]
miR-103/107	miRNA	Human liver and plasma	ob/ob and HFD mice	HepG2	↑	Caveolin-1	Insulin receptor signaling	Reduces insulin sensitivity	Potential experimental target for IR [231]
miR-223	miRNA	Serum of T2DM patients	HFD mice	3T3-L1 adipocytes	↑	GLUT4	PI3K/Akt	Glucose uptake and adipocyte metabolism	Potential biomarker of metabolic dysfunction [232]
miR-146a	miRNA	PBMCs and plasma	db/db mice	Macrophages	↓	IRAK1, TRAF6	NF-κB	Anti-inflammatory regulator	Potential predictor of diabetic inflammation [233]
miR-802	miRNA	Liver biopsies	HFD mice	HepG2	↑	HNF1B	AMPK–FOXO1	Hepatic IR	Potential Experimental target [234]
miR-143	miRNA	Adipose tissue	HFD mice	3T3-L1 adipocytes	↑	ORP8	AKT signaling	Adipogenesis and IR	Associated with obesity-related T2DM [235]
lncRNA GAS5	lncRNA	Serum and PBMCs	Diabetic mice	INS-1 cells	↓	miR-222/PTEN axis	PI3K/Akt	β-cell protection and insulin secretion	Promising circulating biomarker [236]
lncRNA MIAT	lncRNA	Serum and retina	STZ rats	Müller cells	↑	miR-150/VEGF	VEGF signaling	Diabetic retinopathy	Marker of microvascular complications [237]
lncRNA TUG1	lncRNA	Kidney biopsies	db/db mice	HK-2 cells	↓	PGC-1α	AMPK/SIRT1	Renal metabolic regulation	Potential biomarker of diabetic nephropathy [238]
lncRNA ANRIL	lncRNA	Peripheral blood	Diabetic mice	Endothelial cells	↑	VEGF, EZH2	NF-κB	Vascular inflammation	Potential predictor of vascular complications [239]
circANKRD36	circRNA	Peripheral blood mononuclear cells	—	THP-1 macrophages	↑	miR-145	NF-κB	Inflammatory activation	Circulating inflammatory biomarker [240]
circRNA_0054633	circRNA	Peripheral blood	—	—	↑	Predicted miRNA network	Insulin signaling-related	Glucose metabolism	Potential biomarker for prediabetes and T2DM [241,242]

## Data Availability

No new data were created or analyzed in this study. Data sharing is not applicable to this article.
